# A review of ‘medical’ knowledge of epilepsy amongst isiZulu-speaking patients at a regional hospital in KwaZulu-Natal

**DOI:** 10.4102/phcfm.v7i1.789

**Published:** 2015-07-08

**Authors:** Zamir A. Gilani, Kantharuben Naidoo, Andrew Ross

**Affiliations:** 1 Department of Family Medicine, Prince Mshiyeni Memorial Hospital, South Africa; 2 Department of Family Medicine, University of KwaZulu-Natal, South Africa

## Abstract

**Background:**

Epilepsy is a common disorder in South Africa and the literature indicates that many patients do not access treatment. The reasons are complex and include a poor knowledge about causes, symptoms, diagnosis and treatment (medical knowledge). This study aimed to assess the medical knowledge of isiZulu-speaking people with epilepsy (PWE) who attend a combination regional and district hospital in the eThekwini district in KwaZulu-Natal Province.

**Method:**

This was a prospective, cross-sectional, descriptive study. Data were collected using a validated data collection tool for assessing the medical knowledge of PWE and analysed descriptively.

**Results:**

The questionnaires were completed by 199 PWE, with the general level of schooling being low and half being unemployed. Knowledge around causes, symptoms, diagnosis and treatments was good, but there were significant gaps in knowledge that may affect morbidity and mortality.

**Discussion:**

The findings will serve as a useful guide to develop both preventive and educational interventions to enhance knowledge around the causes and treatment of epilepsy in this population. It is important that such interventions also consider family and healthcare providers.

**Conclusion:**

There were considerable gaps in the medical knowledge of isiZulu-speaking PWE's, indicating the need for an educational intervention to improve their understanding of epilepsy. Further research is needed-using a range of tools to ensure that the data is reliable and valid–if the results are to be generalisable to the rest of the province and South Africa.

## Abstrait

Examen des connaissances ‘médicales’ sur l’épilepsie chez les patients de langue zouloue d’un hôpital régional au KwaZulu-Natal.

**Contexte:**

L’épilepsie est un trouble courant en Afrique du Sud et la littérature montre que de nombreux patients n’ont pas accès aux traitements. Les raisons en sont complexes et comprennent une mauvaise connaissance des causes, symptômes, diagnostics et traitements (connaissances médicales). Cette étude avait pour but d’évaluer les connaissances médicales des personnes de langue zouloue atteintes d’épilepsie (PAE) qui fréquentent à la fois les hôpitaux régionaux et de district dans la région d’eThekwini dans la Province du KwaZulu-Natal.

**Méthode:**

C’est une étude prospective, descriptive et transversale. Les données ont été collectées au moyen d’un outil de récolte des données validé pour évaluer les connaissances médicales des PAE, puis analysées de façon descriptive.

**Résultats:**

Les questionnaires ont été remplis par 199 PAE, avec un faible niveau de scolarisation et dont la moitié était au chômage. Les connaissances sur les causes, les symptômes, le diagnostic et les traitements étaient bonnes, mais il y avait des lacunes importantes sur les connaissances affectant la morbidité et la mortalité.

**Discussion:**

Les résultats serviront de guide utile pour mettre au point des interventions à la fois préventives et éducatives afin d’améliorer les connaissances des causes et le traitement de l’épilepsie dans cette population. Il est important que ces interventions examinent aussi la famille et les prestataires de soins de santé.

**Conclusion:**

Il y a des lacunes considérables dans les connaissances médicales des PAE de langue Zouloue, ce qui indique un besoin en matière d’intervention éducative pour améliorer leur compréhension de l’épilepsie. Il faudra pousser les recherches– au moyen de toutes sortes d’outils pour s’assurer que les données sont fiables et valides − si l’on veut généraliser les résultats dans le reste de la province et en Afrique du Sud.

## Introduction

Epilepsy is a common, chronic neurological disorder that affects approximately 69 million people worldwide.^1^ It affects individuals of all ages, ethnicities, socio-economic class and geographic location.^2^The prevalence is estimated as 1% of the total global population, and there are over 13 million people with epilepsy (PWE)residing in Africa.^1^Epilepsy is particularly common in low-income countries, where the prevalence is more than twice that of high-income countries, possibly due to the higher incidence of risk factors.^3^

The World Health Organization (WHO) estimates that, in Africa, 80% of PWE do not receive treatment, particularly those who are disadvantaged and marginalised, despite effective treatment options being available.^4^ Reasons why African patients, in particular, do not access or receive care and treatment are complex and interrelated and include: limited financial resources, poor patient-provider communication, a lack of social support for those with the condition, as well as patient, societal and healthcare provider factors.^5^In addition, patients and their families may lack basic ‘medical’ knowledge about causes of the disorder, as well as its symptoms, diagnosis and treatment. A lack of ‘medical’ knowledge was demonstrated in a large door-to-door survey in Senegal amongst 4500 people, where half reported that epilepsy is caused by evil spirits, a third said that it is contagious, and a quarter reported that traditional therapy was better than western treatment.^5^A lack of knowledge, including medical knowledge, underpinned by poor literacy, has been shown to contribute directly to problems associated with poor medicine regimen compliance.^5^As a consequence, PWE often have a lower quality of life than people with other chronic illnesses.^6^A systematic literature review in 2000 reported the mortality rate of PWE in Africa to be 6.2 times greater than that of the general population, with treatment-avoidance behaviour being found to be a significant contributory factor.^6^

Societal beliefs around epilepsy vary from country to country, and may negatively influence health-seeking strategies. For example, people may not seek treatment if epilepsy is not seen as a condition that can be treated by western medicine.^6^In addition, PWE can be socially stigmatized, and may deny their disease when they are described by derogatory labels attached to epilepsy, such as ‘mad pig disease.’^7^

A study amongst healthcare workers in Zambia in 2007 found that their poor knowledge about epilepsy and its treatment affected access to appropriate management for PWE.^8^ This study recommended that health care workers should receive ongoing education to improve their diagnosis and treatment skills. In addition, educational programmes should address underlying negative attitudes or mistaken knowledge amongst health care workers that may worsen the stigma associated with epilepsy.^8^

In South Africa, although the burden of epilepsy is largely unknown, it is likely to be large, as a 2005 study of children in a large rural community demonstrated a prevalence of 6.7/1000.^9^ In another South African study 2341 adults were screened and the prevalence of epilepsy was found to be 13.8/1000, with only 14.7% taking any regular anti-epileptic treatment.^10^ The study reported that managing and treating epilepsy were greatly influenced by knowledge, cultural attitudes and beliefs, which varied widely.^10^ Participants in both studies were mainly black Africans, which is significant, as many people in this historically disadvantaged population, where there is ongoing stigma around epilepsy, visit traditional healers and respect their opinions.^10^

The high level of epilepsy in South Africa, specifically amongst the African population, highlights the need to establish to what extent this could be affected by their ‘medical knowledge’, specifically in KwaZulu-Natal (KZN). The aim of this study was therefore to assess the ‘medical ‘knowledge of isiZulu-speaking PWE receiving treatment at a combination regional and district hospital in the eThekwini District of KZN. Medical knowledge considers issues such as cause, symptoms, diagnostic methods and treatment of epilepsy. This study will provide information about isiZulu-speaking PWE, and will assist in developing a comprehensive, standardised package for medical education around epilepsy.

## Method

The study design was prospective, descriptive and cross-sectional, and was conducted at an urban-based epilepsy clinic at a combination regional and district hospital in the eThekwini District of KZN Province. Patients seen at this clinic consist of those referred from Primary Health Care (PHC) clinics when their epilepsy is difficult to control, as well as those who live around the hospital. At the start of this study, the clinic had a total of 2200 registered patients, with an average of 456 being seen on an appointment-basis each month. All patients are reviewed by a doctor at the epilepsy clinic on each visit, and those who are seizure free after three months are referred back to the PHC clinic closest to their home (where such a clinic is available).

The study population included patients 18 years or older attending the clinic, with those affected by mental retardation being excluded. A sample size of 199 patients, which represented 44% of the monthly average number of patients attending the clinic was selected. This sample size was considered adequate for a small descriptive cross-sectional study.^11^ To reduce selection bias, every third PWE was invited to participate until the sample size was reached. Files were marked to ensure that patients did not participate more than once. Data collected were from 25 June 2013 to 20 August 2013.

The aim of the study was explained to potential participants by a research assistant who was recruited and supervised by the researcher. The research assistant provided clarity on the questions where required, explained the ‘Study Information Sheet’, and obtained written consent from all participants (consent forms were available in both English and isiZulu, with isiZulu being the first language of most PWE in this context). Participants were requested to self-complete two questionnaires, and the research assistant was available to help those who required assistance. The first questionnaire (Annexure A)obtained demographic details, including level of education, home language, employment status, duration of epilepsy, and whether they were receiving a social grant from the state.

The second data questionnaire(Annexure B) was based on an internationally-validated questionnaire for assessing medical knowledge of PWE – The Epilepsy Knowledge Profile--General (EKP-G).^12^ The EKP-G consists of 55 true/false items (34 medical knowledge items, 21 social knowledge items) that were selected by a range of experts in the field of epilepsy and is considered to be objective, sensitive and unambiguous in its assessment of medical knowledge levels in relation to epilepsy.^12,13^ The questionnaire has also been used in international studies and, in 2003, was found to be useful in comparing the medical knowledge of PWE between countries.^13^

In our study, only the questions regarding medical knowledge were presented to participants in both English and isiZulu. The collected data were entered into SPSS (Chicago, Illinois) and analysed descriptively. The EKP-G was piloted by asking the research assistant and five PWE selected from the epilepsy clinic to complete the questionnaire. The resulting analysis indicated that it was understood and instructions to complete it were clear. Having undergone a rigorous process of validation in other contexts and piloted in the KZN context, the EKP-G was considered to be a valid measurement tool for PWEs’ medical knowledge.

Approval for the study was given by the hospital management, the KZN Provincial Department of Health and the Biomedical Research Ethics Committee of University of KZN (BE 158/11). Permission to use the EKP-G questionnaire was obtained from the instrument's developer.

## Results

A total of 199 PWE completed the questionnaires and the results are presented in two sections: demographic profile and EPK-G results.

### Demographic profile

Of the 199 respondents, 86 were male (43%), and the ages ranged from 18 to 69 years. The majority (83%) did not complete their formal school education, 9% never attended school, and only 5.5% attained a post-school qualification. A summary of the findings is presented in Table 1.

**TABLE 1:** Level of education.

Variable	Number	Percent
No schooling	18	9.0
Grade 1-4	51	25.6
Grade 5-7	26	13.1
Grade 8-11	70	35.2
Matric completed	23	11.6
Higher than Matric	11	5.5
Total	199	100.0

**FIGURE 1:**
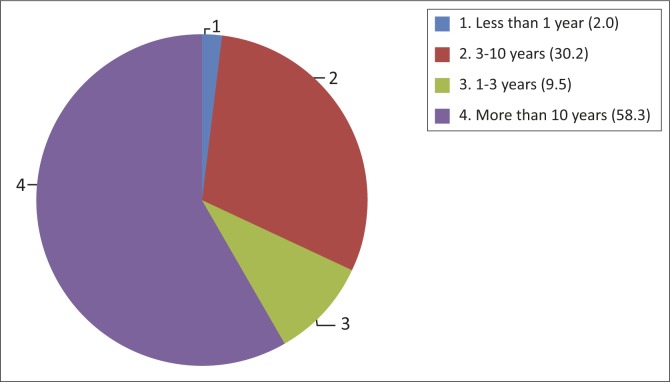
Percentage of patients and duration of epilepsy (years).

Most participants spoke isiZulu as a first language, 2% spoke English and 3.5% spoke another language. Half (53%) reported that they were currently unemployed, 40.7% had never been employed, and 51% were receiving a social grant from the state. Just over half (58%) had been diagnosed with epilepsy for more than 10 years (Figure 1).

A regression equation that considered three variables (highest level of education achieved, duration of epilepsy and highest level of medical knowledge) showed a correlation between level of education and medical knowledge.

### Results of EKP-G questionnaire

The following four tables summarize the results of the EPK-G questionnaire (the number of participants and percentage who answered that question correctly). The findings are presented as participants’ medical knowledge on: (a) cause of epilepsy (Table 2), (b) symptoms (Table 3), (c) diagnosis (Table 4) and (d) treatment (Table 5).

**TABLE 2:** Knowledge of causes of epilepsy (*n* = 199).

Question	Correct Answer	Correct Responses	
		Number	Percent
Epilepsy is always caused by brain damage	No	67	34
Epilepsy is not infectious	Yes	161	81
Certain forms of brain damage always causes epilepsy	No	13	07
An epileptic seizure can be described as an abnormality in the function of nerve cells of the brain	Yes	174	87
Too much alcohol make seizures more likely	Yes	179	90
Stress may cause some seizures	Yes	189	95

**TABLE 3:** Knowledge of symptoms of epilepsy (*n* = 199).

Question	Correct Answer	Correct Responses	
		Number	Percent
Epilepsy is a symptom of mental illness	No	70	35
All people with epilepsy have similar symptoms	No	106	53
All people with epilepsy lose consciousness during epilepsy	No	33	17
Some seizures may last a matter of seconds and not be noticed by others	Yes	147	74
Some people get a warning or a feeling just before a seizure	Yes	165	83
Most seizures result in brain damage	No	16	08

**TABLE 4:** Knowledge of diagnosis of epilepsy (*n* = 199).

Question	Correct Answer	Correct Responses	
		Number	Percent
An EEG can be useful to help diagnose epilepsy	Yes	174	87
If an EEG is abnormal this is a definite sign of epilepsy	No	48	24
An EEG is designed to detect electrical activity from the brain	Yes	175	88
A normal EEG means that you do not have epilepsy	No	93	47

**TABLE 5:** Knowledge of treatment of epilepsy (*n* = 199).

Question	Correct Answer	Correct Responses	
		Number	Percent
For most people doctors can treat epilepsy effectively with drugs	Yes	126	63
All those who start drugs for their epilepsy have to take them for life	No	8	4
Increasing the dose of anti-epileptic drugs increases the chance of side effects	Yes	164	82
In order for anti-epileptic drugs to be successful, they must be taken regularly	Yes	192	96
If you forget to take anti-epileptic drug for a day, it is usually OK to take two doses together	Yes	22	11
Blood samples can be used to detect the concentration of anti-epileptic drugs in the system	Yes	187	94
People who are taking a combination of anti-epileptic drugs are more likely to have side effects than those takingonly one drug	Yes	93	47
Most peoples’ seizures are well controlled soon after starting regular drug treatment	Yes	179	90
It is always helpful to take extra doses of anti-epileptic medication when not feeling well	No	177	89
If seizures stop with anti-epileptic drugs, this means that your epilepsy is cured	No	175	88
Few people with a diagnosis of epilepsy are on anti-epileptic drugs	No	139	70
There is no need to continue taking your anti-epileptic drugs if your seizures stop	No	164	82

When asked about the causes of epilepsy (Table 2), responses indicate that the majority of participants (81%) knew that epilepsy is not an infectious disease, and that it can be exacerbated by alcohol (90%) and stress (95%). It was encouraging that the majority (87%) believed epilepsy to be caused by an abnormality in the function of the nerve cells of the brain, as this suggests that they believed there was a medical cause for epilepsy and did not associate it with witchcraft or supernatural causes.

Regarding their knowledge about epilepsy symptoms, a majority (74%) knew that these varied depending on the type and that epilepsy may not always be associated with generalised seizures (e.g., temporal lobe epilepsy may be associated with absence seizures lasting seconds that are not noticed). A significant proportion (83%) knew of the association between an aura and epilepsy, which could encourage them to seek help when they felt that a seizure was imminent (Table 3.).

Participants generally had a good knowledge about the use of EEG in epilepsy diagnosis (87%), as indicated in Table 4.

**FIGURE 2:**
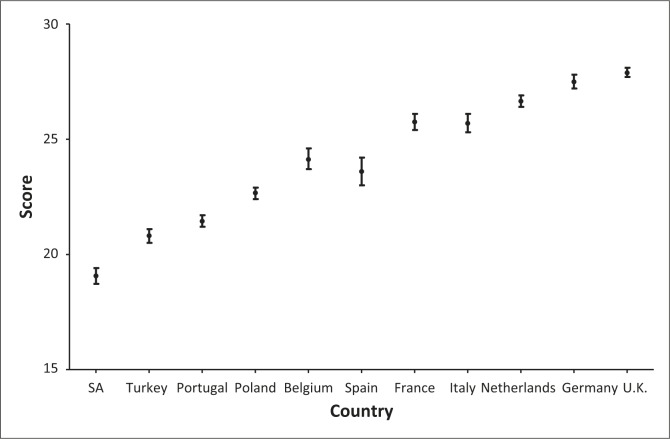
Comparison of current study with 2003 European study.

The majority believed that all PWE would have to take drugs for the rest of their lives (96%), whilst a third (37%) did not know that epilepsy can largely be treated and controlled with drugs. However, this latter finding contradicts the answers to two similar questions: in order for anti-epileptic drugs to be successful they must be taken regularly (96%) and most peoples’ seizures are well controlled soon after starting drugs (90%).The reasons for this discrepancy in responses need to be investigated further. Most PWE knew they must continue medication despite an absence of seizures (82%). (Table 5; Figure 2)

## Discussion

The 199 participants represented a substantial proportion of the average monthly attendance at the clinic. The majority of participants were black and isiZulu-speaking, which represents the demographics of the population served by this combination hospital. Many participants were unemployed, which may affect their ability to access treatment regularly. Of concern was the over 50% of participants receiving disability grants, despite epilepsy being a managed condition enabling people to work if they take appropriate medication. This suggests that either control is so poor that patients are unable to work, that stigma makes it difficult for patients with epilepsy to find work, or that health care practitioners fill in disability grant forms more for social than for medical reasons. With high levels of unemployment in South Africa and the type of work available to PWE often being restricted,^14^ obtaining a disability grant may be a more attractive option than looking for work. Future research could explore why so many patients receive social grants, and their effect on adherence, seizure control, and clinic attendance on this vulnerable population.

Education levels were generally low, and whilst the regression model showed a correlation between level of education and medical knowledge, the number of people in this category was small and the type of post-school education unknown. This finding is in contrast to a study amongst black African university students in South Africa, which showed no association between a higher level of education and correct knowledge about epilepsy.^15^ The study indicated that the students had limited knowledge regarding epilepsy, with the participants believing that a PWE is a witch or wizard, recommending that they be isolated for the safety of others.^15^

As most participants had suffered with epilepsy for more than a year (98%), their knowledge regarding the condition would be expected to be good, as they would have had access to education about the disease during their regular visits to the epilepsy clinic for follow-up. It was encouraging that most knew that epilepsy is associated with abnormal electrical activity in the brain, which is in contrast to an Ethiopian study where only a quarter of participants had the correct knowledge about the causes of epilepsy.^16^ However, it was of concern that most respondents in this study believed that seizures always result in brain damage.

Most participants knew that epilepsy is not an infectious disease, and this knowledge may assist in reducing the stigmatization and social isolation of PWE. However, a difficult concept to explain to PWE may be that some infections do lead to epilepsy, such as neurocysticercosis (NCC), which is a common cause of epilepsy in KZN. A study in the Eastern Cape Province in South Africa illustrated that 100% of participants (*n* = 2431) had no knowledge of NCC, despite it being a common cause for epilepsy in the area.^17^ A literature review did not identify any studies regarding the knowledge of isiZulu PWE about NCC in KZN. A further study amongst this population may indicate whether or not they have any knowledge of NCC, and a prevention campaign based on these findings could be implemented.

It was of concern that two thirds (65%) associated epilepsy with a mental disease, this possibly being due to information provided by health care professionals (HCP's). In a study in Zambia, more than 50% of the HCP's who participated considered epilepsy to be a form of mental illness.^8^ This highlights the need for educational interventions to be extended to HCP's to ensure that they have the correct knowledge about the condition and manifest the appropriate attitude towards PWE, which can then be cascaded down to patients and the broader society.

That most participants had knowledge of EEG may reflect the fact that they were diagnosed at a regional hospital where the equipment was available. Any education programme which is developed should, however, consider the general lack of EEG testing equipment in South Africa, particularly at PHC facilities^17^and inform patients of the need to be referred to regional facilities should this test be considered necessary.

It was encouraging to know that most PWE knew that drugs should be continued despite the absence of seizures and that medication must be taken regularly, as prescribed by the healthcare provider. However, it was of concern that the overall levels of medical knowledge compared poorly with PWE in Europe, with low levels of knowledge highlighting the need for urgent educational initiatives to change this situation.

The findings from this study may be of particular interest to healthcare providers who are in a unique and influential position to enhance medical knowledge as well as dispel misconceptions and myths around epilepsy. The findings regarding the gaps in knowledge about epilepsy causes, symptoms, diagnosis and treatment could be used to guide the design of a targeted educational programme for PWE and health care workers. The literature indicates that education is an effective tool that can be used to enhance knowledge about the disease and highlight the importance of adhering to a specific medical and lifestyle regimen.^18^

To validate the findings and to make them more generalisable, additional measures need to be taken to triangulate the findings, namely that data could be collected from other sites using a variety of data collection instruments and methods. Medical knowledge of PWE has been gathered using several other data collection tools such as the Epilepsy Knowledge Questionnaire (EK-Q), which is a ten item questionnaire.^16^ Other data collection instruments would be useful to consider other aspects of medical knowledge, for example a family's knowledge regarding what to do when their relative has a seizure. A review of the factors associated with societal and healthcare workers ‘knowledge regarding epilepsy would add to the information available to address problems associated with poor ‘medical’ knowledge of isiZulu-speaking PWE receiving treatment in KZN.

## Limitations to the study

A limitation to the study concerns some ambiguity associated with the questions on the data collection instrument, as well as the lack of a ‘don’t know’ option. However, as this was a validated tool used previously in other countries, a decision was taken not to change the questions. A way forward would be to triangulate findings by collecting data on medical knowledge using an alternative data collection instrument. It is also possible that the study was biased towards literate patients, as those unable to read may have been less likely to participate. However, an attempt was made to overcome this by the use of a research assistant to help these patients.

## Conclusion and recommendations

This study provided useful information about the medical knowledge of patients with epilepsy who attend the epilepsy clinic at this combination hospital in the eThekwini District of KZN. The results could be used to design an education programme for isiZulu-speaking PWE. In this context, an education programme should consider issues around causes, diagnosis, prevention and treatment, and include enhancing their knowledge about infections, namely NCC and HIV, which can result in epilepsy. Increasing medical knowledge may lead to reduced stigmatization and isolation, and may encourage patients and family to see the condition as a chronic medical disorder.
